# Dissection in biology education compared to alternative methods in terms of their influence on students’ emotional experience

**DOI:** 10.3389/fpsyg.2023.1138273

**Published:** 2023-05-24

**Authors:** Lisa-Maria Kaiser, Sabrina Polte, Tim Kirchhoff, Nadine Großmann, Matthias Wilde

**Affiliations:** ^1^Faculty of Biology, Biology Didactics, Bielefeld University, Bielefeld, Germany; ^2^BiProfessional, Bielefeld University, Bielefeld, Germany; ^3^Faculty of Mathematics and Natural Sciences, Institute for Biology Education, University of Cologne, Cologne, Germany

**Keywords:** biology education, dissections, disgust, interest, boredom, emotions, video, anatomical model

## Abstract

**Introduction:**

Dissecting animal organs is a method of biology teaching that offers a direct and authentic view into morphological structures and enables hands-on activity and multisensory experiences. However, the dissection process is often associated with certain (negative) emotions that might hinder successful learning. One such emotion that is particularly common during dissection is disgust. Experiencing disgust can negatively affect emotional experiences. Consequently, alternatives for dissection in biology lessons are being sought.

**Methods:**

In this study, the method of dissection is compared with two common methods of teaching the anatomy of the mammalian eye: watching a video and working with an anatomical model. The focus of the comparison is on the influence on the following emotional qualities of experience: perceived disgust, perceived interest, well-being and boredom. Two hundred and eighteen students (*M*_age_ = 14.19, *SD*_age_ = 1.02 years, 52% female) from secondary schools in Germany participated in a two-hour lesson on the anatomy of the mammalian eye using one of the three aforementioned teaching methods.

**Findings:**

Our results show that perceived disgust was higher for the dissection group than in the ones that worked with a video or a model. We found that dissecting and watching a video led to a similar level of interest, well-being, and boredom. The anatomical model was perceived as less disgusting but more boring than the dissection. The detailed videos of a dissection seem to offer similar positive emotional experiences when compared to dissecting in class and may be an alternative approach when teachers have concerns about performing a real dissection.

## 1. Introduction

One of the main tasks of biology education is to enable students to deal with living beings and to gain a sophisticated insight into the structure and function of organisms and one’s own body (Ministerium für Schule und Weiterbildung des Landes Nordrhein-Westfalen, 2019). The understanding of the anatomy and morphology of living beings can be well illustrated by original objects, including not only living animals and plants but also original visual objects such as zoological specimens or parts of animals such as organs (Kattmann, 2020). Both working with living animals and dissecting animal organs in biology lessons are strongly associated with emotions that can impact students’ learning (Randler et al., 2013; Randler, 2021). Positive emotional experiences such as well-being and interest can have a positive effect on learning (Gläser-Zikuda et al., 2005; Krapp, 2007; Hummel and Randler, 2012; Pekrun, 2014), while negative emotions such as boredom, disgust, or anxiety can have a negative effect on learning processes (Randler et al., 2005; Randler, 2021).

Due to the nature of the subject, biology teachers and their students are probably more often confronted with the negative emotion of disgust than teachers of other subjects (Gropengiesser and Gropengiesser, 1985; Randler et al., 2013; Randler, 2021). Especially when dissecting animal organs, students may experience strong feelings of disgust (Holstermann et al., 2009; Randler et al., 2013). Studies have shown that perceived disgust during dissection can compromise other emotional and motivational qualities of experience such as students’ state of interest, perceived self-efficacy (Holstermann et al., 2009, 2012), and motivation (Randler et al., 2013) and thus negatively affect learning. This is one reason why alternative methods to dissection should be examined with regard to their impact on students’ emotional experience and their relevance for biology teaching.

In this study, the method of dissection is compared with two common alternative methods of teaching the anatomy of the mammalian eye, namely watching a video and working with an anatomical model. The following emotional qualities of experience were focused on in the comparison: disgust, perceived interest, well-being, and boredom.

## 2. Theory

### 2.1. Dissection and working with original objects in biology classes

Dissecting animal organs is a teaching method that offers a direct and authentic view into morphological structures. As a hands-on activity, it enables both methodological learning and understanding the process of scientific inquiry (Bowd, 1993). Thus, dissection is a way to offer students first-hand experiences called *Primärerfahrungen* in German (Klingenberg, 2014; Kattmann, 2020) in biology classes. *Primärerfahrungen* entail that students have immediate contact with the learning object and can interact with it directly (Klingenberg, 2014; Kattmann, 2020). The German term *Primärerfahrungen* must be distinguished from the term “primary experience” as defined by Dewey (1995), which considers a holistic approach during one’s unreflected first contact with a learning object. Through immediate interaction with the original object, *Primärerfahrungen* can lead to higher interest in the learning materials (Hummel and Randler, 2012; Klingenberg, 2014). Another characteristic of *Primärerfahrungen* is learning with multiple sensory and emotional perspectives (Klingenberg, 2014). By contrast, *Sekundärerfahrungen* are characterized by greater distance and abstraction from the original and do not allow direct interaction with the original object (Tunnicliffe and Ueckert, 2007). *Sekundärerfahrungen* can be provided in the form of replicas, videos, anatomical models, pictures, or texts.

*Primärerfahrungen* often allow students to also engage in hands-on activities, meaning learning through one’s own practical experience and active engagement with the learning object. When performing hands-on activities, students work on and investigate the natural object by using scientific instruments and methods (Holstermann et al., 2010). In addition to acquiring subject-specific methodological skills, hands-on methods and *Primärerfahrungen* can promote students’ interest and motivation (Hummel and Randler, 2010; Swarat et al., 2012; Wilde et al., 2012; Klingenberg, 2014). Furthermore, hands-on methods support the acquisition of scientific inquiry skills and scientific knowledge (Caglak, 2017). Teachers value the more in-depth understanding of anatomy and function as well as the 3D experience combined with the haptic experience (Donaldson and Downie, 2007; Zemanova, 2022; see also *Primärerfahrungen*).

Despite these advantages, methodological alternatives to dissections in the classroom such as videos or models are also applied (i.e., more *Sekundärerfahrungen*; see Strauss and Kinzie, 1991; Kinzie et al., 1993; de Villiers and Monk, 2005). One reason to do so is that a dissection is much more time-consuming to prepare and perform than using alternatives (de Villiers and Monk, 2005). Moreover, ethical concerns play an important role in how dissections are handled. The killing of animals for school lessons and the dissection itself are viewed critically by many students (Stanisstreet et al., 1993; Donaldson and Downie, 2007). However, it must be distinguished whether a whole animal is dissected that has been killed specifically for the teaching purposes or if single organs such as lungs or eyes are dissected, which are usually derived from slaughterhouse waste products (Donaldson and Downie, 2007). When implementing such dissections, affective issues need to be considered (see Bowd, 1993). In a study by Zemanova (2022), teachers stated that disgust was the main reason why students did not want to participate in dissection.

In summary, dissection is a teaching method (Bowd, 1993), that might trigger both positive and negative emotional experiences (Holstermann et al., 2009, 2012). Disgust and further emotional qualities of experience are considered in the following section.

### 2.2. Emotional experiences in biology lessons

For students, everyday school life provides a variety of emotions that can influence their learning (Schutz and DeCuir, 2002; Schutz et al., 2006). From a theoretical point of view, emotions are complex and multidimensional constructs (Hascher, 2010). They consist of physiological, cognitive, expressive, and motivational components, and they also inherit an affective core. Affective experience can be classified as positive or negative valence (Frenzel et al., 2020). The motivational influence of emotions lies in promoting positive experiences and avoiding negative states of mind (Abele-Brem and Gendolla, 2000). Furthermore, emotions comprise a state and a trait condition (Frenzel et al., 2020). However, in our study, we focus solely on the state condition, that is the *emotional experience* within the learning situation. From a neurobiology perspective, emotional experiences play a key role in terms of cognitive processing and learning. In brief, emotional experiences can be connected to higher levels of attention and enhanced memorizing (Tyng et al., 2017). Learning situations might trigger intense positive or negative emotions in students (Pekrun and Hofmann, 1999). Negative emotional experiences can lead students to try to avoid a learning situation, whereas positive ones can increase their motivation to learn (Bindra, 1969; Pekrun et al., 2002). Randler (2021) applied the concept of learning emotions to the specific conditions of biology education. In this context, the emotion disgust has an outstanding relevance for biology teaching, especially when working with living/dead animals or dissecting organs (see, e.g., Holstermann et al., 2009; Randler et al., 2012a). However, Holstermann et al. (2012) found that disgust during a dissection does not necessarily dominate the emotional experience and may co-occur with other (desirable) variables such as students’ interest. Therefore, students’ emotional experience during dissections and alternative methods should be viewed holistically in terms of different positive and negative qualities. According to Randler et al. (2011) perceived interest, well-being, and boredom are important learning emotions that should be considered in addition to disgust. In our study, this operationalization allows to *exemplarily* investigate positive emotional qualities of experience, that is students’ perceived interest and well-being, as well as complementary negative emotional qualities, such as disgust and boredom simultaneously. The following sections introduce these four emotional qualities of experience separately.

#### 2.2.1. Disgust

Disgust is considered a negative emotion and is one of the most intense defensive feelings in humans (Ekman and Friesen, 1975; Menninghaus, 1999). It is accompanied by a strong physical reaction which is characterized, for example, by turning away from the disgust-evoking object, an aversion to touch it, or by being repulsed up to the point of retching and vomiting (Ekman and Friesen, 1975). Disgust is an emotion with a variety of potential triggers (Tybur et al., 2009). Tybur et al. (2009) therefore categorize disgust into three different types: pathogenic disgust, sexual disgust, and moral disgust. Disgust for animals and for the organs of dead animals is assigned to pathogenic disgust (Tybur et al., 2009; Prokop and Randler, 2017). Although these defensive feelings are natural protective responses (Curtis et al., 2004; Oaten et al., 2009; Prokop et al., 2010), when students show exaggerated disgust, it can strongly influence biology lessons (Gropengiesser and Gropengiesser, 1985). The emotion disgust can have a negative impact on students’ interest and intrinsic motivation (Holstermann et al., 2009; Randler et al., 2013; Kaiser, et al., in press) and, in turn, their learning (Prokop and Fančovičová, 2017).

#### 2.2.2. Interest

Currently, there are different approaches either conceptualizing interest as a motivational (see, e.g., the person-object-theory of interest; Krapp, 1999, 2007) or an emotional construct (see, e.g., Gläser-Zikuda et al., 2005; Ainley and Ainley, 2011; for an overview see Renninger and Hidi, 2011). From a motivational point of view, interest is defined as a relationship between a person and an object (Krapp, 1992). An interest-driven activity is characterized by a high perceived value of the object (value-related component), a positive emotional quality of experience during the activity (emotional component; Krapp, 1999), as well as the endeavor to learn more about the object (cognitive component; Prenzel, 1988). Moreover, interest is either an enduring motivational disposition (individual interest), or a psychological state that arises within a specific situation (situational interest; Krapp, 2002). Due to this specific psychological state of “being interested” and the positive experience described by the aforementioned components, interest also has an emotional core. Randler et al. (2011) and Gläser-Zikuda et al. (2005) consider interest to be a cognitive-emotional construct. This conceptualization focuses on the respective “appraisal process during the state of interest” in terms of its positive or negative valence (“affective experience”; Renninger and Hidi, 2011, p. 171). In this study, we follow this view of interest as situation-specific emotional experience. Perceived interest might foster students’ willingness to engage in a situation and promotes sustainable learning (Ainley and Ainley, 2011; Renninger and Hidi, 2011).

#### 2.2.3. Well-being

There is no uniform definition of the state of subjective well-being. However, there is agreement that positive emotions such as enjoyment represent the core component of well-being (Diener, 2000; Götz et al., 2004). It is insufficient that no negative emotions occur to experience well-being, but well-being includes the experience of positive emotions (Frenzel et al., 2020). Some definitions focus on the affective dimension of well-being and conceptualize it as a balance of positive and negative emotions that leads to a state of life satisfaction as a positive disposition (Diener and Larsen, 1993; Diener et al., 1999; Lucas, 2016). Accordingly, perceived well-being within a situation is not *one* emotion but a superordinate state of emotional experience that processes several emotions. In relation to school teaching, this means that experiencing enjoyment in class, for example, can lead to positive feelings and well-being. Studies have shown that well-being in biology classes not only depends on the teaching materials but also on the topic of the lesson (Randler, 2021). Students’ perceived well-being in class is a complex phenomenon that is influenced by a variety of factors such as classroom climate and teacher support (Clement, 2010). While interest here emphasizes more the cognitive dimension, well-being refers to the affective dimension of enjoyment during the lesson (Randler et al., 2011). Thus, although interest and enjoyment often occur together, they represent complementary qualities of positive emotional experience (Ainley and Ainley, 2011; Renninger and Hidi, 2011).

#### 2.2.4. Boredom

In contrast to anger or disgust, boredom is usually more quietly expressed, and the teaching process is less disturbed (Pekrun et al., 2010). Boredom is a relatively weak negative emotional state and is usually directly related to an activity (Götz et al., 2007). Even though some student-centered teaching methods such as experimentation or learning at workstations have been shown to counteract boredom, these measures alone are not enough to avoid the occurrence of this emotion (Schaal and Bogner, 2005). Boredom is characterized as a lack of value in the learning situation and the perception that time slowly passes (Pekrun et al., 2010). The activity is perceived as unimportant by the students (Pekrun et al., 2010). Thus, the state of boredom is more than the lack of interest, as it is actively experienced as a negative state and is therefore often associated with avoidance-tendencies. Boredom can correlate negatively with learning performance (Randler et al., 2005; Randler, 2009).

## 3. Hypotheses

Dissections trigger strong emotions such as disgust (Holstermann et al., 2009, 2012). As an alternative to dissections, videos and anatomic models can be used to provide students the same content. Since students have no direct physical contact to the dead materials and other disgust-triggers such as unpleasant smells in these two cases, we hypothesized dissections to be perceived as more disgusting than videos and models. Thus, we tested the following hypothesis:

*H1*: The students who dissect a pig’s eye report more *perceived disgust* than the students who watch a video of a dissection (a) or who work with anatomical models (b).

There are several other emotions that might have an impact on students’ learning (Pekrun and Hofmann, 1999). According to Randler et al. (2011), there are three emotional qualities of experience that are particularly important in the context of biology learning: perceived interest, well-being, and boredom. The emotion disgust correlates negatively with students’ interest and intrinsic motivation (Holstermann et al., 2009, 2012; Randler et al., 2013; Kaiser et al., in press). Regarding these considerations, we furthermore assume that students who engage in a dissection, a potentially “disgusting” method (see, e.g., Holstermann et al., 2009), show a lower degree of positive emotional qualities of experience than students watching a dissection-video or working with anatomic models.

*H2*: The students who dissect a pig’s eye report less *perceived interest* than the students who watch a video of a dissection (a) or who work with anatomical models (b).

*H3*: The students who dissect a pig’s eye report less *well-being* than the students who watch a video of a dissection (a) or who work with anatomical models (b).

In addition to these positive qualities, we examined the students’ boredom during the lesson. As a hands-on method, dissections provide various options of engagement and self-activity (Holstermann et al., 2010), which should counteract boredom. Videos and anatomic models, on the other hand, cannot be influenced by the students and provide less options for interactions. Thus, we tested the following hypothesis:

*H4*: The students who dissect a pig’s eye report less *boredom* than the students who watch a video of a dissection (a) or who work with anatomical models (b).

## 4. Methods

### 4.1. Sample

In the current study, 218 students from two secondary schools in Germany participated. Ninety-nine students (45%) visited a *Gymnasium* (a secondary school type preparing students for higher education ending after the 12th or 13th grade) and 119 students (55%) visited a *Realschule* (another secondary school type ending after the 10th grade and geared towards preparing students for further vocational training). Both schools were located in the federal state of North Rhine-Westphalia. On average, the students were 14.19 (*SD* = 1.02) years old. The distribution of girls and boys was balanced (52% female). The specific distribution of the students referring to the respective grades is illustrated in Table 1. Approximately half of the students have had prior experience with dissection, either by dissecting on their own (28%) or by being a part of a team and passively watching while other students dissected (18%). The other students have had no prior experience with dissection (54%).

**Table 1:** Distribution of the students, their average age, and the proportion of girls depending on the different grades.

Grade	No. students	Average age (mean and standard deviation)	Proportion of girls (%)
8	99	13.38 (0.53)	47
9	43	14.23 (0.52)	61
10	76	15.17 (0.78)	52

### 4.2. Design

We used a quasi-experimental design with two measuring time points to examine our hypotheses (Tolmie et al., 2011; Döring and Bortz, 2016). Between these measuring points, the students attended a two-hour teaching unit about the anatomy of mammalian eyes. This lesson was divided into two parts: (1) text work providing students with content and specific topic-related vocabulary and (2) an explorative phase in which the students examined these structures in detail. In the respective treatments, we varied this last phase into three treatments with one dissecting a pig’s eye (D-treatment), the next watching a video of a dissection (V-treatment), and the last working with an anatomical model (M-treatment). In the D-treatment, the students dissected a pig’s eye according to examination instructions.

If used as a teaching method, dissections inherit two qualities: First, it provides realistic *Primärerfahrungen* with an original object (Kattmann, 2020). Second, as a hands-on method, it provides students the opportunity to be active and explorative (Holstermann et al., 2009). We let the students choose whether they wanted to do the dissection or work with an anatomic model instead. Across all classes, five students did not participate in the dissection and were consequently removed from the sample for the analysis. In the V-treatment, we designed a video showing the dissection of a pig’s eye. The video had the same structure as the dissection instruction and, thus, provided the same content. Moreover, the video allowed the students to deal with a realistic depiction of the original object. There was no physical contact with the dead materials and potentially disgusting smells. Although the scientific inquiry process was visualized, there was no hands-on examination, and the video provided no possibilities for interaction (except for pausing the video). In the M-treatment, the students dealt with an anatomic model of an eye. The students could explore the eye by dissembling the model. In addition, the students received a reader which provided the same structure and information as the dissection instruction and the video. Accordingly, all students received the same content information. In contrast to the last two methods, an anatomical model is an abstract and reduced representation of the original object (Kattmann, 2020). A summary of the key characteristics of each treatment and the respective subsample information are presented in Table 2. We assigned the classes within a school randomly to the three treatments.

**Table 2:** Illustration of the respective treatment and subsample characteristics.

		D-treatment	V-treatment	M-treatment
Treatment characteristics	Method	Dissection of a pig’s eye	Watching a video of a pig’s eye dissection	Working with an anatomical model of an eye
	Object	Original	Real depiction (film)	Abstraction
	Type of experiences	*Primärerfahrung*	*Sekundär erfahrung*	*Sekundär erfahrung*
	Hands-on opportunities	Yes	No	Yes^a^
Specific sub-sample characteristics	Subsample size	*n* = 92	*n* = 61	*n* = 57
	Number of classes	4	3	3
	Age	*M* = 14.09 years *SD* = 0.91 years	*M* = 14.28 years *SD* = 1.06 years	*M* = 14.26 years *SD* = 1.12 years
	Gender distribution	59% female	39% female	53% female

a Self-active, but without hands-on experience with the original natural object.

To assess the students’ emotional experience, we used a quantitative survey with retrospective self-reports. Approximately one week before the start of the intervention, we assessed students’ level of disgust regarding dissections. Furthermore, we asked the students about the extent of their prior experiences with dissections. The situational emotional qualities of experience (well-being, interest, boredom, and disgust) were measured at the end of the lesson.

### 4.3. Measures

The quantitative data was collected by using standardized questionnaires. All scales were measured using a five-point rating scale from *not true at all* (0) to *absolutely true* (4).

#### 4.3.1. Disgust regarding dissection

We measured disgust towards dissection using a 9-item scale for *disgust regarding dissections* (“I am disgusted by dissecting animal organs.”). The scale was based on a scale for animal disgust (see Wilde et al., 2018) and was adapted and complemented to dissections (see Kaiser et al., in press). The questionnaire included items on disgust perception in direct terms and on sensory perception towards dissection such as tactile, visual, and olfactory stimuli that can trigger disgust (Miller, 1997; Petrowski et al., 2010; Liuzza, 2021). Internal consistency was found to be good (Cronbach’s α = 0.85).

#### 4.3.2. Emotional experience

The emotional experience during dissection were assessed using the *situational emotion short scale* (Randler et al., 2011). This questionnaire includes the subscales *interest*, *well-being*, and *boredom* with three items each. The subscale *interest* refers to a subject-topic-relationship and maps the importance and utility of the teaching topic (e.g., “I found that topic important.”). The scale showed adequate internal consistency (Cronbach’s α = 0.75). The subscale *well-being* refers to the emotion enjoyment and a generally positive feeling during the lesson (e.g., “The lesson pleased me.”). The subscale showed good internal consistency (Cronbach’s α = 0.87). The subscale *boredom* includes subject-related boredom and lack of attention during the lesson (e.g., “I felt bored.”). The subscale showed satisfying internal consistency (Cronbach’s α = 0.80). The situational emotion short scale was extended by an additional subscale assessing students’ *perceived disgust* during the lesson with three items (e.g., “I found the topic of this lesson disgusting.”). The items are based on the wording of the other items of the situational emotion short scale (Randler et al., 2011). The original adjectives were replaced by the specific disgust adjectives from the Differential Affect Scale (Merten and Krause, 1993). The internal consistency was found to be good (Cronbach’s α = 0.89).

### 4.4. Statistics

The main goal of our study was to compare students from the dissection treatment (D-treatment) in terms of different indicators of the emotional quality of experience with the alternative methods video (V-treatment) and anatomical model (M-treatment). Thus, we conducted Analysis of Variances (ANOVA) and planned contrast analysis (Field, 2018). In this context, we tested whether the requirements for the ANOVA, such as normal distribution and homogeneity of variance (Tolmie et al., 2011; Field, 2018), were met. As the variables *perceived disgust*, *perceived interest*, and *well-being* did not show variance homogeneity, we used robust alternatives (Field, 2018). All calculations were performed with *IBM SPSS Statistics 28*.

#### 4.4.1. Preliminary analysis

As students’ prior motivational disposition may have an impact on their situational emotional experience, we controlled for potentially confounding factors. Thus, we used a single-factor ANOVA to compare the students in the three treatments concerning their prior disgust regarding dissections. In addition, we controlled for students’ prior disgust regarding dissections at the different grade levels by using a one-factor ANOVA. Because prior studies showed negative relationships between disgust and other motivational and emotional variables such as interest (Holstermann et al., 2012), motivation (Randler et al., 2013), and flow-experience (Polte and Wilde, 2018), as well as interdependencies between the respective emotions (Diener and Iran-Nejad, 1986), we also evaluated intercorrelations (Pearson’s correlations) between all subscales.

#### 4.4.2. Hypotheses testing

As we tested several related variables within the same sample, we performed a multivariate analysis of variance (MANOVA; see Field, 2018). Thus, we assessed an overall test for differences throughout all variables (Field, 2018). To investigate our hypotheses, we used simple contrasts (Bühner and Ziegler, 2009; Field, 2018) to compare students from the D-treatment (comparison category) respectively with students from the V-and M-treatment regarding their *perceived disgust* (H1), *perceived interest* (H2)*, well-being* (H3), and *boredom* (H4). In case of variance heterogeneity, we used robust parameters. The effect-sizes (*r_effect size_*) were calculated according to Sedlmeier and Renkewitz (2008).

## 5. Results

### 5.1. Preliminary analysis

First, we controlled for differences between the respective groups before the start of the intervention. We did not find any significant differences between the students of the different treatments in terms of their prior disgust towards dissections (*F*(2, 117.85) = 0.65, *p* = 0.190). Moreover, we did not find differences between the different grades in terms of students’ prior disgust regarding dissections (*F*(2, 109.30) = 0.03, *p* = 0.973). Furthermore, we found theory-conform correlations between all subscales (see Table 3). Disgust correlated negatively with positive variables such as interest and well-being as well as slightly positive with boredom. Perceived interest correlated with well-being and moderately negatively with boredom. Analogously, boredom and well-being correlated moderately negatively.

**Table 3:** Intercorrelations (Pearson’s correlation coefficient, bivariate) throughout all treatment groups.

	1	2	3	4
1. Perceived disgust	1.00	−0.51^*^	−0.52^*^	0.30^*^
2. Perceived interest		1.00	0.68^*^	−0.48^*^
3. Well-being			1.00	−0.58^*^
4. Boredom				1.00

Significant correlations (*p* < 0.05) are marked with an *.

### 5.2. Hypotheses testing

To test the aforementioned hypothesis, we compared the teaching method dissection to alternative methods in terms of students’ emotional experience. The overall test of the MANOVA revealed statistically significant differences between at least some of the three teaching methods: dissecting, watching a video, or working with anatomical models (Wilk’s Λ = 0.70, *F*(8, 378) = 10.12, *p* < 0.001, η^2^_p_ = 0.17). Accordingly, further analyses for each dependent variable separately are appropriate. However, further contrast analyses revealed only some specific differences between the D-treatment and the other two treatments. The descriptive statistics and results of the respective contrast analysis for all subscales are presented in Table 4.

**Table 4:** Illustration of the descriptive parameters and results of the contrast analyses.

Subscale	Descriptive parameters Mean (Standard deviation)			Simple contrast analyses *t*-statistic, *p*-value, and effect size (*reffect size*)	
	D-treatment	V-treatment	M-treatment	D-vs. V-treatment	D vs. M-treatment
Perceived disgust	1.09 (1.11)	0.73 (0.82)	0.40 (0.59)	***t* (137.95) = 2.23, *p* < 0.05, *r***_***effect size***_ **= 0.19**	***t* (125.98) = 4.70, *p* < 0.001, *r***_***effect size***_ **= 0.39**
Perceived interest	2.62 (0.93)	2.74 (0.62)	2.40 (0.77)	*t* (138.17) = 0.87, *p* = 0.384, *r_effect size_* = 0.07	*t* (128.42) = −1.50, *p = 0.*138, *r_effect size_* = 0.13
Well-being	3.12 (0.88)	3.20 (0.53)	2.91 (0.74)	*t* (134.30) = 0.70, *p* = 0.485, *r_effect size_* = 0.06	*t* (127.78) = −1.51*, p* = 0.133, *r_effect size_* = 0.13
Boredom	0.55 (0.68)	0.80 (0.73)	1.16 (0.96)	*t* (124.27) = 2.10, *p* = 0.061, *r_effect size_* = 0.18	***t* (90.20) = 4.05, *p* < 0.001, *r***_***effect size***_ **= 0.30**

Significant comparisons are highlighted bold.

Regarding *perceived disgust*, contrast analysis showed significant differences between the treatments. Students in the D-treatment reported more perceived disgust than students in the V-treatment. The effect size for this comparison indicates a small effect (*r_effect size_* = 0.19; Cohen, 1988). Furthermore, students in the D-treatment reported more perceived disgust than students in the M-treatment (Table 4). Students in the M-treatment showed the lowest values regarding their perceived disgust. In this context, the effect size indicates a moderate effect (*r_effect size_* = 0.39; Cohen, 1988). Regarding the descriptive parameters, it is noticeable that the mean values in the D-treatment are accompanied with a relatively high standard deviation compared to the mean values in the V-and M-treatment. Regarding the positive emotional qualities *perceived interest* and *well-being*, contrast analysis revealed no significant differences (Table 4). The students from the D-treatment did not report significantly lower values of perceived interest and well-being compared to students from the V-and M-treatment. We investigated students’ *boredom* in addition to the aforementioned positive qualities. Contrast analysis showed that students in the D-treatment reported significantly less boredom than the students in the M-treatment (Table 4). The effect size (*r_effect size_* = 0.30) indicates a medium effect (Cohen, 1988). However, we did not find significant differences between the D-and the V-treatment.

## 6. Discussion

The aim of this study was to compare dissections with alternative methods (working with videos or anatomical models) in terms of students’ emotional qualities of experience (perceived disgust, perceived interest, well-being, and boredom). The results of the study confirmed our first hypothesis. The students who dissected a pig’s eye during the lesson were more disgusted than students who watched a video of a dissection (H1a) or who worked with an anatomic model (H1b). While dissections are *Primärerfahrungen* and offer a multi-sensory experience, the video of a dissection as well as the anatomical model have a greater distance to the original (Kattmann, 2020). With both teaching methods, no direct interaction with the original object is possible and the options for sensory experience are limited. In the case of the video of the dissection of a pig’s eye, a very detailed view of the original is offered that has the potential to evoke disgust (Tolin et al., 1997; Lang et al., 1999). However, when watching the video, some sensory experiences such as the perceived unpleasant smell and the haptic experiences are missing. Both perceptions can trigger or intensify feelings of disgust in addition to the visual impression (Petrowski et al., 2010). The anatomical model is a more abstract representation of the original and thus has a larger distance to the original. Hence, it is not surprising that an abstract representation is perceived as being less disgusting. Nevertheless, it must be noticed that the average perceived disgust was, compared to the scale range (0 to 4), relatively low (D-Treatment: *M* = 1.09, *SD* = 1.11) and the standard deviation relatively high. This indicates a rather low perceived disgust, but high variation regarding the subjective ratings.

Since previous studies (see, e.g., Holstermann et al., 2009; Randler et al., 2013; Polte and Wilde, 2018; Kaiser et al., in press) showed that motivational variables such as interest, flow, and self-efficacy are negatively related to disgust, we assumed that a method that clearly evokes disgust (see H1) affects the development of interest and other positive emotional qualities such as well-being and thus ultimately leads to lower emotional qualities than methods that evoke less disgust. While intercorrelations (Table 3) also show a negative relationship between disgust and perceived interest and well-being as well as a positive relationship to boredom, the findings of our study contradict this assumption on a treatment level. The students in the dissection treatment did not generally report poor qualities of emotional experiences. It should be noted that the methods dissecting, watching a video, and using the model resulted in similar levels of the positive emotional qualities of perceived interest (H2a) and well-being (H3a). The methods dissection and watching a video both allow for a very detailed view of the original object. While the video lacks the possibility of hands-on experience, the method working with an anatomical model offers this possibility but no sensory experience with the original object. While some studies have shown that hands-on activities have a positive effect on students’ interest (Swarat et al., 2012; Wilde et al., 2012), this effect did not become apparent in our study. The mere method or hands-on activity does not necessarily lead to higher interest (Holstermann et al., 2010; Dohn, 2013). Thus, the topic of the lesson, the mammalian eye, might be more important than the method for the development of perceived interest (see Randler et al., 2005). After all, the topic of the lesson was the same in all three treatments and the interest in human biology topics is particularly pronounced among students in the 8th to 10th grades (Löwe, 1987; Holstermann and Bögeholz, 2007). Ritchie et al. (2016) also showed that it is possible to be very disgusted in lessons and still be very interested in the topic at the same time. A possible explanation is that disgust might also increase attention to the subject matter (van Hooff et al., 2013). Increased attention, in turn, can increase perceived interest, as the catch component mentioned in the development of interest suggests (Mitchell, 1993; Krapp, 1998). Thus, contrary to our second hypothesis, the students in the D-treatment did not show a lower level of perceived interest than the students in the V-and the M-treatment.

This was also evident in the results for our third hypothesis on well-being. Despite the differences in perceived disgust, the subjective well-being was found to be equally high in all treatment groups (Table 4). Apparently, all students enjoyed the lesson on the mammalian eye regardless of the method used. Enjoyment of learning is an important component of subjective well-being in the classroom and can increase students’ interest (Pekrun, 2014; Sutter-Brandenberger et al., 2018). The high degree of well-being in all treatment groups could also be related to the fact that all three methods were different from regular biology lessons and were therefore perceived as new and interesting. The novelty of a learning situation can be influenced by various factors such as new teaching methods, learning locations, or even teachers (Dohn, 2013). In our study, both dissecting and watching a dissection video represented an unusual learning situation for students in biology class. Moreover, in all treatment groups, the lessons were conducted by student teachers from the university and not by the regular biology teacher. Novelty can motivate and capture students’ attention (Mitchell, 1993) and promote situational interest. Well-being, in turn, can characterize the positive affective qualities of being interested. The results of the intercorrelations also show that interest and well-being are strongly positively correlated with each other (Table 3).

Our fourth hypothesis was only partially confirmed. The students who dissected showed significantly less boredom than students who worked with an anatomical model (H4b). As a hands-on method, dissection offers many opportunities to become active, which counteracts boredom (Holstermann et al., 2010; Minkley et al., 2017). The anatomical model offers fewer opportunities for interaction and is more distant from the original object due to the stronger degree of abstraction. The video offers no opportunities for hands-on experiences and such passive methods are usually perceived as being more boring compared to student-active methods (Randler et al., 2005; Minkley et al., 2017). On the other hand, dissecting was perceived as more disgusting than watching a video or working with a model. Similar to the positive emotional qualities of experience (H2a and H3a), no statistical differences were found for boredom between the D-Treatment and the V-Treatment in our study (H4a). Both methods allow a view of the original perceived disgusting object and, as already pointed out, disgust can also lead to increased attention (van Hooff et al., 2013). Increased attention is in itself a characteristic of the psychological state of interest (Ainley et al., 2002), and in the video treatment, enjoyment and interest in the lesson may have outweighed boredom.

Negative emotions do not necessarily interfere with school learning (Strauss and Kinzie, 1991; Hascher and Edlinger, 2009; Pekrun, 2014), and emotions may co-exist in an individual as long as one does not dominate over the others (Diener and Iran-Nejad, 1986; Holstermann et al., 2012). This was also evident in our study. Even though dissection was perceived as significantly more disgusting than the other methods, the perceived interest and well-being in this group was not lower than in the other two groups. On the treatment level, it seems to be confirmed that several positive and negative emotions can be expressed at the same time in a learning situation. However, negative emotions such as disgust do not fundamentally undermine subjective well-being and the perception of interest. The possible co-occurrence of disgust is therefore not a sufficient reason to decide against using dissections as a teaching method. Our study shows that positive emotional experience and low boredom can be highly pronounced despite using a clear disgust-evoking method. Therefore, the differences between classes should always be considered in a sophisticated way. On this basis, it should be decided whether the implementation of a dissection is useful. It is important to let students decide about the extent to which they want to participate in disgust-evoking activities. In addition, less disgusting alternatives should be offered that allow the students to determine the distance to the disgusting object themselves. A well-documented video of a real dissection is a good method to offer a less disgusting alternative. Especially as these can still lead to similar positive emotional experience in the classroom as a real dissection. Working in groups is one more important possibility to reduce students’ stress (e.g., triggered by strong disgust) in learning situations (Minkley et al., 2017). Another approach is to use a combination of the different methods in the classroom. For example, in a study by Akplan and Andre (1999), it was shown that the use of a simulation of a dissection of a frog before the real dissection had a positive effect on the students’ knowledge of anatomy. Thus, a video of dissection could also be used as preparation for the real dissection to familiarize students with the supposedly disgusting learning situation. Holstermann et al. (2010) also found that prior experience in hands-on activities lead to increased interest in biological methods such as dissection. Other studies (Randler et al., 2012b, 2013; Wüst-Ackermann et al., 2018) found that disgust with certain learning objects was reduced through school-based and pedagogically prepared exposure to the object. The reduction of disgust in relation to natural objects can therefore also represent an important learning outcome of biology lessons.

### 6.1. Limitations

Despite our promising results, some limitations need to be addressed. First, emotions and emotional qualities of experience are complex constructs (Frenzel et al., 2020) that also do not always have uniform definitions (Götz et al., 2004; Tybur, 2021). For instance, it is not clear which emotions determine subjective well-being (Götz et al., 2004). Other factors such as the classroom climate and teacher support are also important for the development of subjective well-being at school (Clement, 2010). Three different components also contribute to the development of situational interest (Krapp, 1999). A more differentiated view of the individual components could provide more detailed findings. For example, Kaiser et al. (in press) were able to show that there are differences in the effect of the disgust experience on the three components of interest (value-related, cognitive, and emotional).

Second, disgust is a strong negative emotion that can be very stressful for some students and then hinder successful learning (Ekman and Friesen, 1975; Prokop and Fančovičová, 2017). A differentiated look at the students who felt strongly disgusted would also be useful here as particularly strong disgust can correlate with other emotional qualities (Randler, 2009; Randler et al., 2013). A person-centered approach considering disgust simultaneously with other motivational variables with a larger sample could be the aim of further studies. However, this also reveals a difficulty in implementing studies with students who are strongly disgusted. As described above, when dealing with disgust in class, it is important not to force students to participate. However, five students in the D-treatment who were particularly disgusted by dissection did the tasks with an anatomical model and did not participate in the dissection. We excluded these students from the sample because they did not participate in the actual dissection. However, next to these research-methodological considerations, from a practical point of view it must be discussed how disgust might be decreased and how all students can be motivated to engage in the dissection task. One way could be to prepare the students for the actual activity with a video of the dissection (Randler et al., 2007). Possibly some of these students would then decide to participate in the actual dissection in the group.

Third, in the debate about the use of alternatives to dissection, ethical reasons and animal welfare play a particularly important role, which will not be discussed further here. It is important to inform students that the organs they dissect are products of human consumption or waste products from this process (Spernjak and Šorgo, 2017). The animals were therefore not killed for the sake of school lessons, per se. The learning video is also a good alternative from this point of view, as only one organ is used for recording the dissection, but it can serve as a visual tool for many students.

### 6.2. Conclusion

Our study was able to confirm that disgust correlates negatively with positive emotional qualities of experience and negatively with boredom. For students who are strongly disgusted, this negative emotion can predominate over other qualities of experience and impair learning in biology classes. However, this correlation could not be confirmed in the comparison of the methods implemented at the treatment level. Even if disgust correlates negatively with interest, and well-being, this does not mean that methods such as dissection automatically lead to less positive emotional experiences in the teaching unit. In our study, the differences regarding the emotional qualities of experience between the dissecting treatment and the alternative treatments seem to be small. Detailed video recordings of dissection usually elicit a similar emotional response and thus offer a good alternative experience to the real dissection.

## Data availability statement

The datasets presented in this article are not readily available because this study is part of an ongoing research project and qualification works in which further analysis are planned. Requests to access the datasets should be directed to lisa-maria.kaiser@uni-bielefeld.de.

## Ethics statement

The studies involving human participants were reviewed and approved by the Ethik-kommission der Universität Bielefeld. Written informed consent to participate in this study was provided by the participants’ legal guardian/next of kin.

## Author contributions

L-MK developed the study concept and design, recruited the sample, performed most of the statistical analysis, and wrote parts of the methods and hypotheses as well as the results sections. SP wrote the first draft of all other article sections and developed the article concept with L-MK. TK contributed to the development of the study design, performed parts of the statistical analysis, and reviewed the article. NG contributed to the statistical procedure and reviewed and revised the article. MW contributed to the study design and reviewed the article. All authors contributed to the article and approved the submitted version.

## Funding

This project is part of the “Qualitätsoffensive Lehrerbildung,” a joint initiative of the Federal Government and the *Länder* which aims to improve the quality of teacher training. The programme is funded by the Federal Ministry of Education and Research (funding number 01JA1908). The authors acknowledge support for the publication costs by the Open Access Publication Fund of Bielefeld University and the Deutsche Forschungsgemeinschaft (DFG). The authors are responsible for the content of this publication.

## Conflict of interest

The authors declare that the research was conducted in the absence of any commercial or financial relationships that could be construed as a potential conflict of interest.

## Publisher’s note

All claims expressed in this article are solely those of the authors and do not necessarily represent those of their affiliated organizations, or those of the publisher, the editors and the reviewers. Any product that may be evaluated in this article, or claim that may be made by its manufacturer, is not guaranteed or endorsed by the publisher.
